# Short-term spinal cord stimulation vs. pulsed radiofrequency in acute/subacute herpes zoster pain

**DOI:** 10.3389/fmed.2026.1859392

**Published:** 2026-07-30

**Authors:** Chen Li, Xinyu Dou, Wenhui Liu, Chunhua Liu, Qing Shi, Yi Zhang, Yifan Li, Bifa Fan, Peng Mao

**Affiliations:** 1 Department of Pain Medicine, China-Japan Friendship Hospital, Beijing, China; 2 Graduate School, Beijing University of Chinese Medicine, Beijing, China

**Keywords:** postherpetic neuralgia, pulsed radiofrequency, retrospective study, short-term spinal cord stimulation, zoster-related pain

## Abstract

**Context:**

Acute and subacute zoster-related pain (ZRP) may progress to postherpetic neuralgia (PHN), yet comparative real-world evidence for early neuromodulation remains limited.

**Objectives:**

To compare the clinical outcomes of short-term spinal cord stimulation (stSCS) and pulsed radiofrequency (PRF) in patients with acute/subacute ZRP.

**Methods:**

In this retrospective single-center cohort study, 421 patients with acute/subacute ZRP treated with stSCS (*n* = 167) or PRF (*n* = 254) between 2020 and 2023 were included. Outcomes included pain intensity assessed by the Numeric Rating Scale (NRS), treatment response, residual ZRP and PHN incidence, sleep quality assessed by the Athens Insomnia Scale (AIS), activities of daily living (ADL), and complications.

**Results:**

Both treatments were associated with significant pain reduction during follow-up. Compared with PRF, stSCS achieved lower NRS scores at 2 weeks and 1 month (both *p* < 0.001), and higher responder rates at 2 weeks (80.2% vs. 50.8%) and 1 month (92.2% vs. 75.6%; both p < 0.001). At 3 months, responder rates remained higher with stSCS (97.6% vs. 92.9%, *p* = 0.043), whereas by 6 months response rates were similarly high in both groups (98.8% vs. 98.0%, *p* = 0.708). PHN incidence was lower in the stSCS group (7.2% vs. 20.9%, *p* < 0.001). In multivariable adjusted analyses, stSCS remained associated with a lower risk of PHN incidence. Sleep quality improved in both groups, with greater AIS improvement after stSCS (all post-treatment *p* < 0.001), while ADL improvement was comparable. No permanent neurological deficit or device-related infection occurred. Three mild pneumothorax cases after thoracic PRF resolved with conservative management.

**Conclusion:**

Short-term spinal cord stimulation was associated with faster early pain relief, greater sleep improvement, and lower incidences of residual ZRP and PHN than PRF in acute/subacute ZRP.

## 1 Introduction

Herpes zoster (HZ) results from reactivation of latent varicella–zoster virus and affects about 1/3 people over a lifetime, with roughly one million cases annually in the United States (1). The most frequent and debilitating complication is zoster-related pain (ZRP), which may evolve into postherpetic neuralgia (PHN). Published estimates suggest that PHN develops in approximately 5–30% of patients with HZ overall, approximately 12.5% of patients aged ≥50 years, and an even higher proportion of older or high-risk patients (2–4).

Clinically, ZRP progresses through acute, subacute, and chronic phases. PHN is conventionally defined as pain persisting for ≥90 days after rash onset (5). In the acute/subacute phase, when nociceptive and neuropathic mechanisms overlap, peripheral and central sensitization may contribute to pain chronification. This window represents a critical therapeutic opportunity, highlighting the rationale for early and mechanism-targeted interventions (6).

Current standard care primarily includes prompt antiviral therapy—ideally within 72 h of rash onset—alongside analgesics and adjuvant neuropathic agents. While antivirals accelerate lesion healing and modestly alleviate acute pain, they have limited efficacy in preventing PHN (7). Consequently, many patients continue to experience significant pain despite optimized pharmacological management, underscoring the need for interventional strategies.

Among minimally invasive options, pulsed radiofrequency (PRF) applied to the dorsal root ganglion (DRG) achieves nondestructive neuromodulation to suppress ectopic discharges, whereas short-term spinal cord stimulation (stSCS) involves temporary percutaneous leads connected to an external generator for approximately 1–2 weeks (8, 9). The conceptual rationale for stSCS in acute/subacute ZRP is not simply symptomatic analgesia; early dorsal column stimulation may reduce ongoing afferent barrage to the dorsal horn, suppress wide-dynamic-range neuronal hyperexcitability, restore segmental inhibitory balance, activate descending inhibitory systems, and attenuate neuroimmune/glial amplification before pain chronification becomes established (10–12).

Evidence supporting both modalities is accumulating but remains heterogeneous. Retrospective studies have demonstrated meaningful analgesia with stSCS in acute/subacute ZRP, and a randomized trial comparing stSCS with PRF reported superior pain relief and reduced rescue analgesic use in the stSCS group over 1–6 months (13, 14). Chinese expert consensus supports considering stSCS within the first 3 months of herpetic-related neuralgia when pain is severe or refractory (9). Meta-analyses suggest stSCS provides greater analgesic benefit with an acceptable safety profile compared with PRF, although heterogeneity, limited sample sizes, and inconsistent PHN definitions temper definitive conclusions (4, 15). Therefore, the prophylactic or disease-modifying role of early stSCS remains plausible but not proven.

Despite these advances, real-world comparative evidence remains sparse, particularly from single-center cohorts with standardized management pathways during the acute/subacute phase—when interventions may still alter the trajectory toward chronification. To address this gap, we conducted a retrospective, single-center study comparing short-term spinal cord stimulation versus pulsed radiofrequency for acute/subacute herpes zoster pain.

## 2 Methods

### 2.1 General information

**Figure 1:**
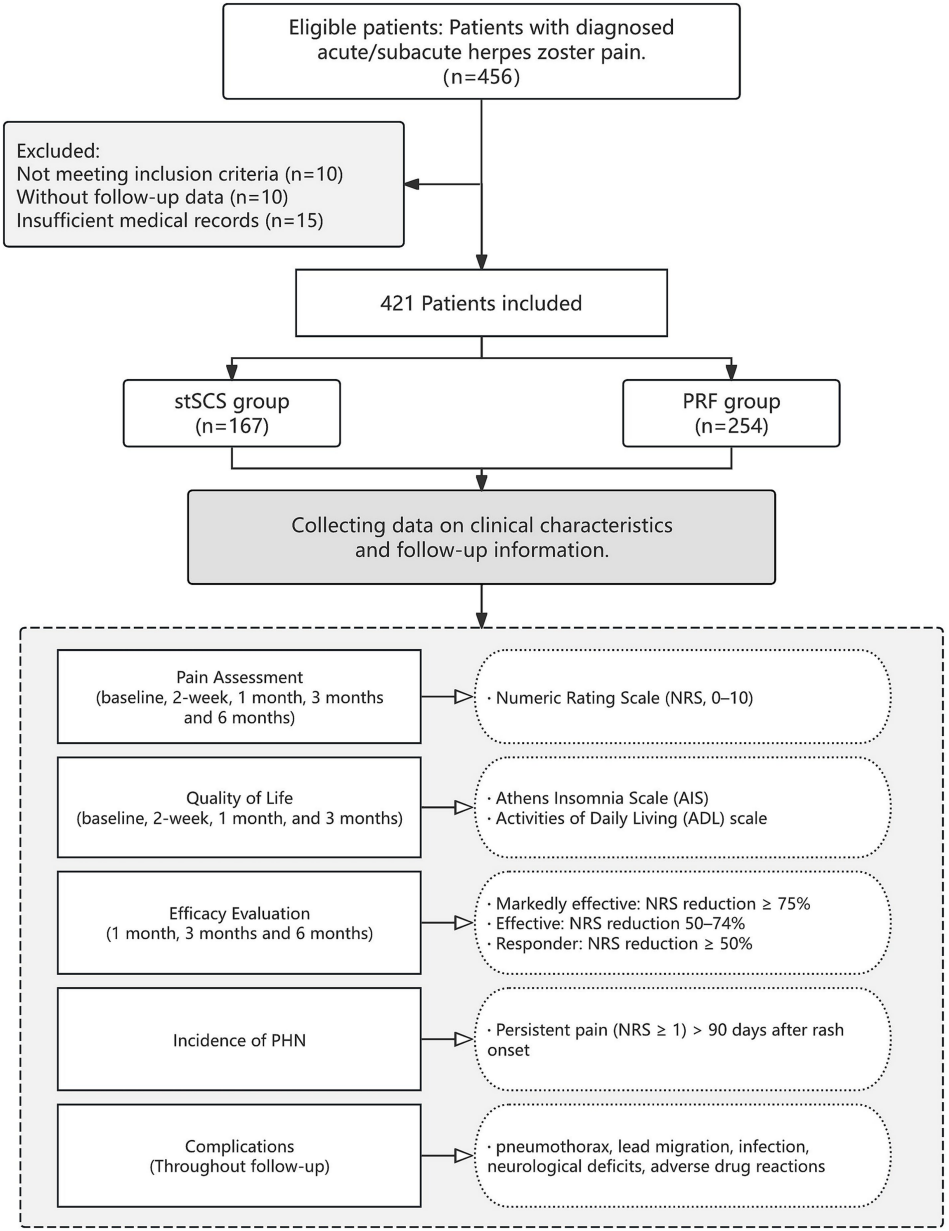
Flowchart of patient screening, enrollment, treatment allocation, and outcome assessment.

This retrospective study was conducted at China-Japan Friendship Hospital, Beijing, China, between December 2020 and December 2023. This study was approved by the Human Ethics Committee of China-Japan Friendship Hospital (2023-KY-343). The technical workflow of this study is illustrated in Figure 1. Patients diagnosed with acute or subacute zoster-related pain who underwent either stSCS or PRF were screened. Demographic characteristics (age, sex, BMI), disease duration, comorbidities, and dermatome involved were collected from the electronic medical records (Table 1).

**Table 1:** Baseline characteristics of the study population.

Characteristic	stSCS group (*n* = 167)	PRF group (*n* = 254)	*p*-value
Age (years)	67.0 ± 9.1	68.9 ± 11.2	0.065
Sex, n (%)			0.134
Male	86 (51.5%)	111 (43.7%)	
Female	81 (48.5%)	143 (56.3%)	
BMI	22.5 ± 3.7	21.9 ± 3.4	0.115
Disease duration (days)	47.7 ± 24.0	47.8 ± 24.5	0.958
Phase			0.425
Acute phase	82 (49.1%)	114 (44.9%)	
Subacute phase	85 (50.9%)	140 (55.1%)	
Baseline NRS	7.0 (6.0, 9.0)	7.0 (6.0, 9.0)	0.455
Baseline AIS score	12.0 (9.0, 15.0)	12.0 (8.0, 15.0)	0.092
Baseline ADL score	65.0 (60.0, 70.0)	65.0 (60.0, 70.0)	0.451
Dermatome involved			
Cervical	34 (20.4%)	58 (22.8%)	0.630
Thoracic	101 (60.5%)	135 (53.1%)	0.160
Lumbosacral	32 (19.1%)	61 (24.1%)	0.280
Comorbidities			
Hypertension	73 (43.7%)	102 (40.2%)	0.481
Diabetes	49 (29.3%)	70 (27.6%)	0.740
Coronary heart disease	23 (13.8%)	46 (18.1%)	0.282
Immune diseases	9 (5.4%)	18 (7.1%)	0.547
Malignant tumors	11 (6.6%)	19 (7.5%)	0.847
Liver/ kidney dysfunction	19 (11.4%)	20 (7.9%)	0.234
Other	11 (6.6%)	10 (3.9%)	0.256

Data are presented as mean ± standard deviation (SD), median (interquartile range), or number (%), as appropriate. *p*-values indicate comparisons between the stSCS and PRF groups.

BMI, body mass index; NRS, Numeric Rating Scale; AIS, Athens Insomnia Scale; ADL, Activities of Daily Living; SD, standard deviation.

Treatment allocation was not randomized. In routine clinical practice, the choice between stSCS and PRF was made through shared decision-making after standardized evaluation by pain physicians, taking into account pain severity and distribution, disease duration, affected dermatome, expected need for broader neuromodulatory coverage, patient preference, tolerance of temporary epidural lead placement and an external pulse generator, procedural risk, cost and resource availability. stSCS was generally considered when rapid and sustained neuromodulation over a broader dermatome was desired, whereas PRF was generally considered when patients preferred a less invasive, shorter procedure or declined temporary lead implantation. This real-world allocation process may introduce selection bias and residual confounding and is therefore explicitly considered in the interpretation of comparative outcomes.

### 2.2 Inclusion and exclusion criteria

#### 2.2.1 Inclusion criteria

1. Age ≥ 18 years.
2. Clinical diagnosis of herpes zoster with moderate-to-severe pain (NRS ≥ 3).
3. Disease duration ≤ 90 days from rash onset (acute: ≤30 days; subacute: 31–90 days).
4. Received either stSCS or PRF as initial interventional therapy.
5. Complete clinical data available and follow-up ≥ 3 months.

#### 2.2.2 Exclusion criteria

1. Patients who received invasive neuromodulation therapy for the current episode before the index intervention.
2. Patients who received crossover intervention or rescue neuromodulation with the alternative procedure within 3 months after the index intervention, including rescue stSCS after PRF or rescue PRF after stSCS.
3. Severe psychiatric disorders or cognitive impairment affecting pain assessment.
4. Coagulation disorders, uncontrolled infection, or contraindications to implantation.
5. Incomplete medical records or loss to follow-up.

### 2.3 Treatment methods

1. Short-Term Spinal Cord Stimulation (stSCS): Under fluoroscopic guidance, percutaneous leads were placed in the epidural space targeting the dorsal columns corresponding to the affected dermatomes. The lead was connected to an external pulse generator and stimulation was maintained for 7–14 days with individualized parameter adjustment (frequency 20–60 Hz, pulse width 200–400 μs, voltage below motor threshold).
2. Pulsed Radiofrequency (PRF): PRF was applied to the affected dorsal root ganglion (DRG) using a radiofrequency generator. Under fluoroscopic guidance, the electrode was positioned adjacent to the DRG. After satisfactory sensory stimulation and negative motor stimulation, PRF treatment was delivered with a pulse frequency of 2 Hz, pulse width of 20 ms, voltage titrated to approximately 45 V or the maximal tolerable voltage, maximum electrode-tip temperature ≤42 °C, and total treatment time of 600 s.

**Figure 2:**
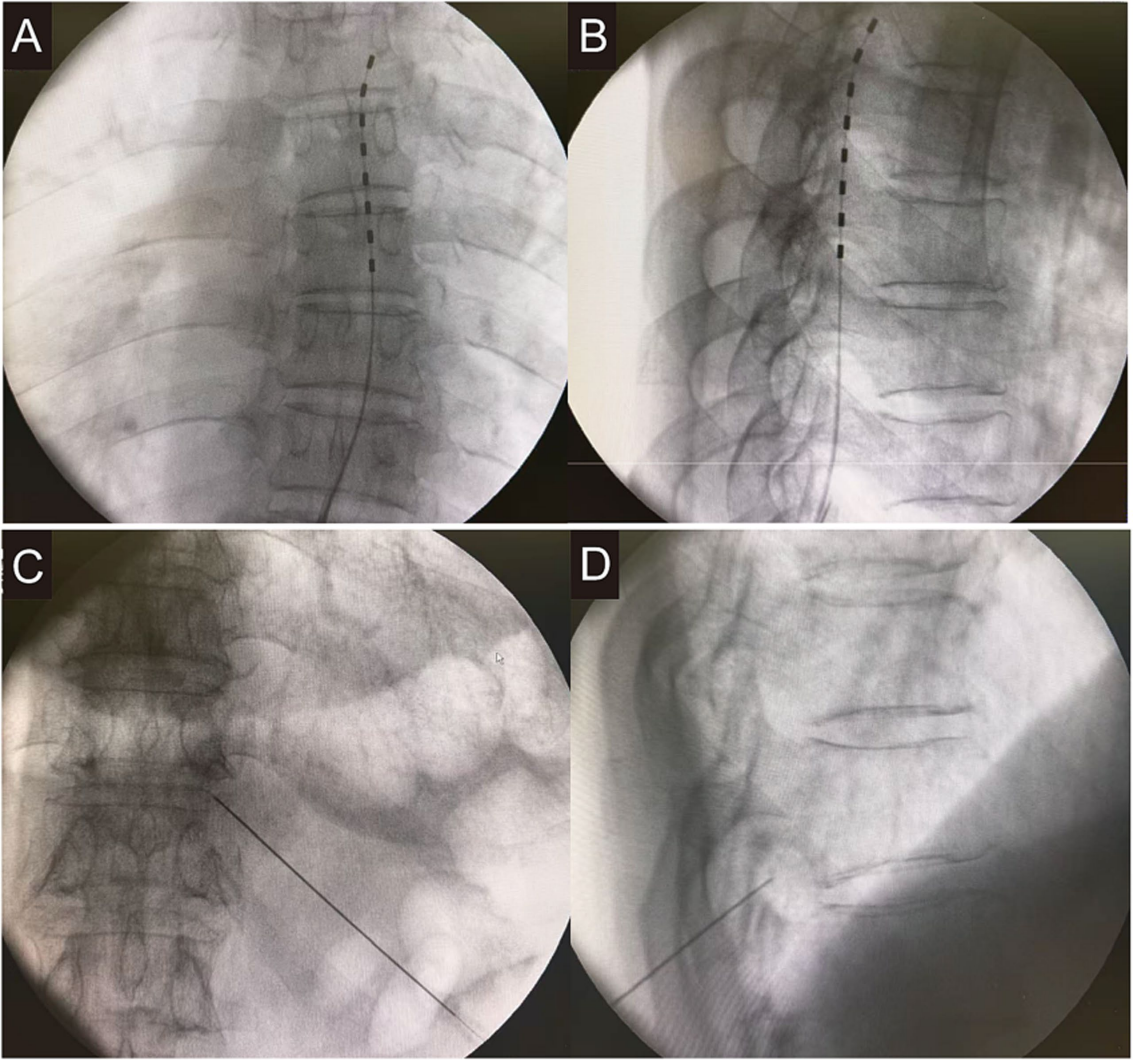
Representative fluoroscopic images of the stSCS and PRF procedures. **(A,B)** Anteroposterior and lateral fluoroscopic views showing epidural lead placement for stSCS. **(C,D)** Anteroposterior and lateral fluoroscopic views showing cannula positioning for PRF targeting the dorsal root ganglion.

Intraoperative fluoroscopic images (anteroposterior and lateral views) showing electrode (Vectris™ 1 × 8 percutaneous lead, Model 977A290; Medtronic, Minneapolis, MN, USA) placement for spinal cord stimulation (Figures 2A,B) and cannula (22-gauge RF cannula with a 10-mm active tip, BNS Medical, Beijing, China) positioning for pulsed radiofrequency targeting the dorsal root ganglion (Figures 2C,D).

All patients in both groups received standardized pharmacological treatment according to the same institutional clinical pathway for acute/subacute zoster-related pain. The routine regimen included antiviral therapy when clinically indicated, gabapentinoids such as pregabalin or gabapentin for neuropathic pain control, non-opioid analgesics and/or opioids according to pain severity, and antidepressant adjuvants such as duloxetine or tricyclic antidepressants when sleep disturbance, anxiety symptoms, or refractory neuropathic pain were present. Rescue analgesics were permitted according to a stepwise protocol based on NRS score and clinical response, and medication adjustments were made by pain physicians during follow-up. The principles of concomitant pharmacological management were identical in the stSCS and PRF groups, although specific agents and doses were individualized according to patient tolerance, comorbidities, contraindications, and treatment response.

### 2.4 Observation indicators

#### 2.4.1 Efficacy evaluation

Treatment efficacy was evaluated according to pain reduction and functional improvement:

1. Markedly effective: ≥75% reduction in NRS score.
2. Effective: 50–74% reduction in NRS score.
3. Responder: Patients achieving an NRS reduction ≥50% (including both markedly effective and effective cases).

#### 2.4.2 Pain assessment

Pain intensity was assessed using the Numeric Rating Scale (NRS) at baseline, 2 weeks, 1 month, 3 months, and 6 months after intervention.

#### 2.4.3 Incidence of residual ZRP and PHN

The incidence of residual ZRP was recorded and defined as any persistent zoster-related pain with an NRS score ≥ 1 at ≥ 90 days after rash onset. This threshold was selected as a sensitive endpoint to capture any residual pain after the acute/subacute phase, including mild residual symptoms. Given that several studies define clinically significant postherpetic neuralgia (PHN) using persistent pain with an NRS score ≥ 3 at ≥90 days after herpes zoster onset, clinically significant PHN was prespecified as a sensitivity endpoint in the present study (16). Accordingly, the incidence rates of both residual ZRP (NRS ≥ 1) and clinically significant PHN (NRS ≥ 3) were evaluated.

#### 2.4.4 Quality of life assessment

Sleep quality was evaluated with the Athens Insomnia Scale (AIS), and functional status with the Activities of Daily Living (ADL) scale. Both were assessed at baseline, 2 weeks, 1 month, and 3 months after treatment.

#### 2.4.5 Complications

Procedure- or device-related complications (e.g., lead displacement, infection, neurological deficits, adverse drug reactions) were documented throughout follow-up.

### 2.5 Statistical methods

All statistical analyses were performed using SPSS version 27.0 (IBM Corp., Chicago, IL, United States). Continuous variables were tested for normality using the Shapiro–Wilk test. Normally distributed continuous variables were expressed as mean ± standard deviation (SD) and compared using the independent-samples *t*-test, whereas non-normally distributed continuous or ordinal variables were presented as median (interquartile range, IQR) and compared using the Mann–Whitney *U* test. Categorical variables were summarized as counts and percentages and compared using the *χ*^2^ test or Fisher’s exact test, as appropriate.

For within-group comparisons across multiple time points, repeated measures of non-normally distributed outcomes (including NRS, AIS, and ADL scores) were analyzed using the Friedman test, followed by *post hoc* pairwise comparisons with the Wilcoxon signed-rank test and Bonferroni correction for multiple testing. For between-group comparisons of longitudinal outcomes at follow-up time points, Mann–Whitney *U* tests were used, and Bonferroni correction was applied within each outcome family to control for multiple comparisons.

Treatment response rates and residual ZRP/PHN incidence were compared between groups using *χ*^2^ analysis or Fisher’s exact test, and effect sizes were expressed as odds ratios (ORs) with 95% confidence intervals (CIs). To account for potential confounding due to the nonrandomized treatment allocation, multivariable logistic regression analyses were performed for persistent pain outcomes, including residual ZRP and PHN incidence.

Subgroup analyses were performed according to dermatome distribution, disease phase, and baseline pain severity. Heterogeneity of treatment effects across subgroups was evaluated using the Breslow–Day test for interaction. All statistical tests were two-tailed, and a *p*-value < 0.05 was considered statistically significant.

## 3 Results

### 3.1 Demographic and clinical characteristics

A total of 421 patients were enrolled, including 167 in the stSCS group and 254 in the PRF group. Baseline characteristics, including age, sex distribution, disease duration, disease phase, baseline NRS, AIS, and ADL scores, affected dermatome, and comorbidities, were comparable between the two groups (all *p* > 0.05, Table 1).

### 3.2 Pain relief

**Figure 3:**
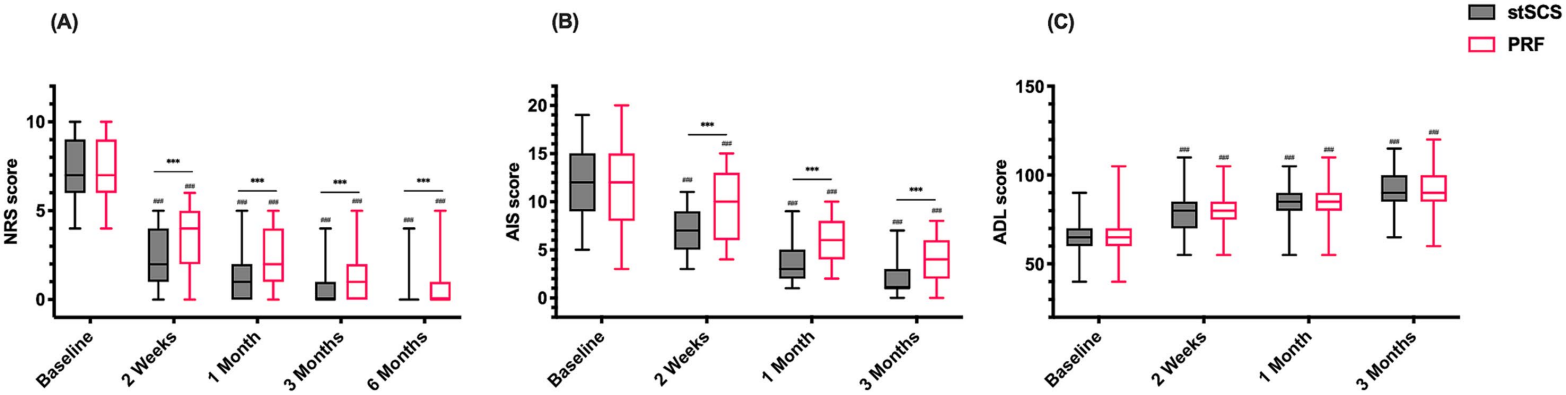
Changes in pain intensity, sleep quality, and functional status during follow-up in the stSCS and PRF groups. **(A)** NRS scores at baseline, 2 weeks, 1 month, 3 months, and 6 months. **(B)** Athens Insomnia Scale (AIS) scores at baseline, 2 weeks, 1 month, and 3 months. **(C)** Activities of Daily Living (ADL) scores at baseline, 2 weeks, 1 month, and 3 months. Boxes indicate the interquartile range, center lines indicate the median, and whiskers indicate the range. ***p < 0.001 for between-group comparisons at the same time point; ^###^*p* < 0.001 versus baseline within the same treatment group.

Both interventions resulted in significant pain relief compared with baseline during follow-up (Figure 3A). As shown in Table 2, patients in the stSCS group experienced a marked reduction in pain intensity, with median NRS scores decreasing from 7.0 (6.0, 9.0) at baseline to 2.0 (1.0, 4.0) at 2 weeks, 1.0 (0, 2.0) at 1 month, 0 (0, 1.0) at 3 months, and 0 (0, 0) at 6 months. Similarly, in the PRF group, median NRS scores declined from 7.0 (6.0, 9.0) at baseline to 4.0 (2.0, 5.0) at 2 weeks, 2.0 (1.0, 4.0) at 1 month, 1.0 (0, 2.0) at 3 months, and 0 (0, 1.0) at 6 months. Although a statistically significant difference was observed at 6 months, the absolute between-group difference was small, and both groups demonstrated near-complete pain resolution.

**Table 2:** Improvement of NRS score after treatment.

NRS Score	stSCS	PRF	*p*-value
Baseline	7.0 (6.0, 9.0)	7.0 (6.0, 9.0)	0.455
2 weeks	2.0 (1.0, 4.0)	4.0 (2.0, 5.0)	<0.001
1 month	1.0 (0, 2.0)	2.0 (1.0, 4.0)	<0.001
3 months	0 (0, 1.0)	1.0 (0, 2.0)	<0.001
6 months	0 (0, 0)	0 (0, 1.0)	<0.001

Data are presented as median (interquartile range). For post-treatment between-group comparisons of NRS scores, Bonferroni correction was applied across four follow-up time points, with an adjusted significance threshold of 0.0125. *p*-values indicate between-group comparisons at each time point. Within-group significance over time is illustrated in Figure 3A; NRS scores at all post-treatment time points were significantly lower than baseline in both groups (all *p* < 0.001).

NRS, Numeric Rating Scale.

Between-group comparisons demonstrated that the stSCS group achieved significantly greater pain reduction than the PRF group at all post-treatment time points (all *p* < 0.001, Table 2), with particularly pronounced differences observed at 2 weeks and 1 month. Within-group significance is illustrated in Figure 3A, with all post-treatment NRS scores being significantly lower than baseline (all *p* < 0.001).

### 3.3 Overall efficacy

As shown in Table 3, stSCS was associated with significantly higher responder rates than PRF at early follow-up time points. At 2 weeks, the responder rate reached 80.2% in the stSCS group compared with 50.8% in the PRF group, corresponding to a markedly increased likelihood of response with stSCS (OR = 3.93, 95% CI 2.50–6.19; *p* < 0.001). This advantage persisted at 1 month (92.2% vs. 75.6%, OR = 3.83, 95% CI 2.03–7.21, p < 0.001) and remained statistically significant at 3 months (97.6% vs. 92.9%, OR = 3.11, 95% CI 1.03–9.35, *p* = 0.043). With respect to treatment intensity, stSCS yielded a substantially higher proportion of markedly effective responses at 2 weeks, 1 month, and 3 months, with odds ratios consistently favoring stSCS (all *p* < 0.001). Conversely, the proportion of patients categorized as “effective” (defined as an NRS reduction of 50–74%) tended to be comparable between groups at early time points and was higher in the PRF group at 3 months, reflecting a shift toward more moderate analgesic responses in the PRF cohort. By 6 months, responder rates were similarly high in both groups (98.8% for stSCS vs. 98.0% for PRF, OR = 1.66, 95% CI 0.32–8.64, *p* = 0.708), indicating convergence of long-term analgesic outcomes despite earlier differences in response magnitude. However, because records of crossover or rescue interventions between 3 and 6 months were incomplete, this apparent convergence should be interpreted cautiously and mainly as a descriptive finding.

**Table 3:** Comparison of effective rates and responders.

Indicator		stSCS	PRF	OR (95% CI)	*p*-value
2 weeks	Markedly effective	76/167 (45.5%)	48/254 (18.9%)	3.58 (2.31–5.55)	<0.001
	Effective	58/167 (34.7%)	81/254 (31.9%)	1.14 (0.75–1.72)	0.597
	Responder	134/167 (80.2%)	129/254 (50.8%)	3.93 (2.50–6.19)	<0.001
1 month	Markedly effective	112/167 (67.1%)	119/254 (46.9%)	2.31 (1.54–3.47)	<0.001
	Effective	42/167 (25.1%)	73/254 (28.7%)	0.83 (0.54–1.30)	0.436
	Responder	154/167 (92.2%)	192/254 (75.6%)	3.83 (2.03–7.21)	<0.001
3 months	Markedly effective	154/167 (92.2%)	168/254 (66.1%)	6.06 (3.25–11.31)	<0.001
	Effective	9/167 (5.4%)	68/254 (26.8%)	0.16 (0.08–0.32)	<0.001
	Responder	163/167 (97.6%)	236/254 (92.9%)	3.11 (1.03–9.35)	0.043
6 months	Markedly effective	155/167 (92.8%)	227/254 (89.3%)	1.54 (0.76–3.12)	0.303
	Effective	10/167 (6.0%)	22/254 (8.7%)	0.67 (0.31–1.46)	0.352
	Responder	165/167 (98.8%)	249/254 (98.0%)	1.66 (0.32–8.64)	0.708
PHN incidence		12/167 (7.2%)	53/254 (20.9%)	0.29 (0.15, 0.57)	<0.001
Adjusted^*^				0.30 (0.14–0.66)	0.003
Residual ZRP incidence		18/167 (10.8%)	71/254 (28.0%)	0.31 (0.18–0.55)	<0.001
Adjusted^*^				0.35 (0.18–0.70)	0.002

Data are presented as number/total number (%). *P*-values indicate comparisons between the stSCS and PRF groups. Odds ratios (ORs) with 95% confidence intervals (CIs) are shown for the effect of stSCS relative to PRF. Markedly effective was defined as an NRS reduction ≥ 75%; effective as an NRS reduction of 50–74%; and responder as an NRS reduction ≥ 50%. PHN was defined as persistent pain with an NRS score ≥ 3 beyond 90 days after herpes zoster onset. Residual ZRP was defined as persistent pain with an NRS score ≥ 1 beyond 90 days after herpes zoster onset. Adjusted^*^, adjusted ORs were derived from separate multivariable logistic regression models for residual ZRP and PHN, with PRF as the reference group.

OR, odds ratio; CI, confidence interval; NRS, Numeric Rating Scale; PHN, postherpetic neuralgia; ZRP, zoster-related pain.

### 3.4 Incidence of residual ZRP and PHN

**Figure 4:**
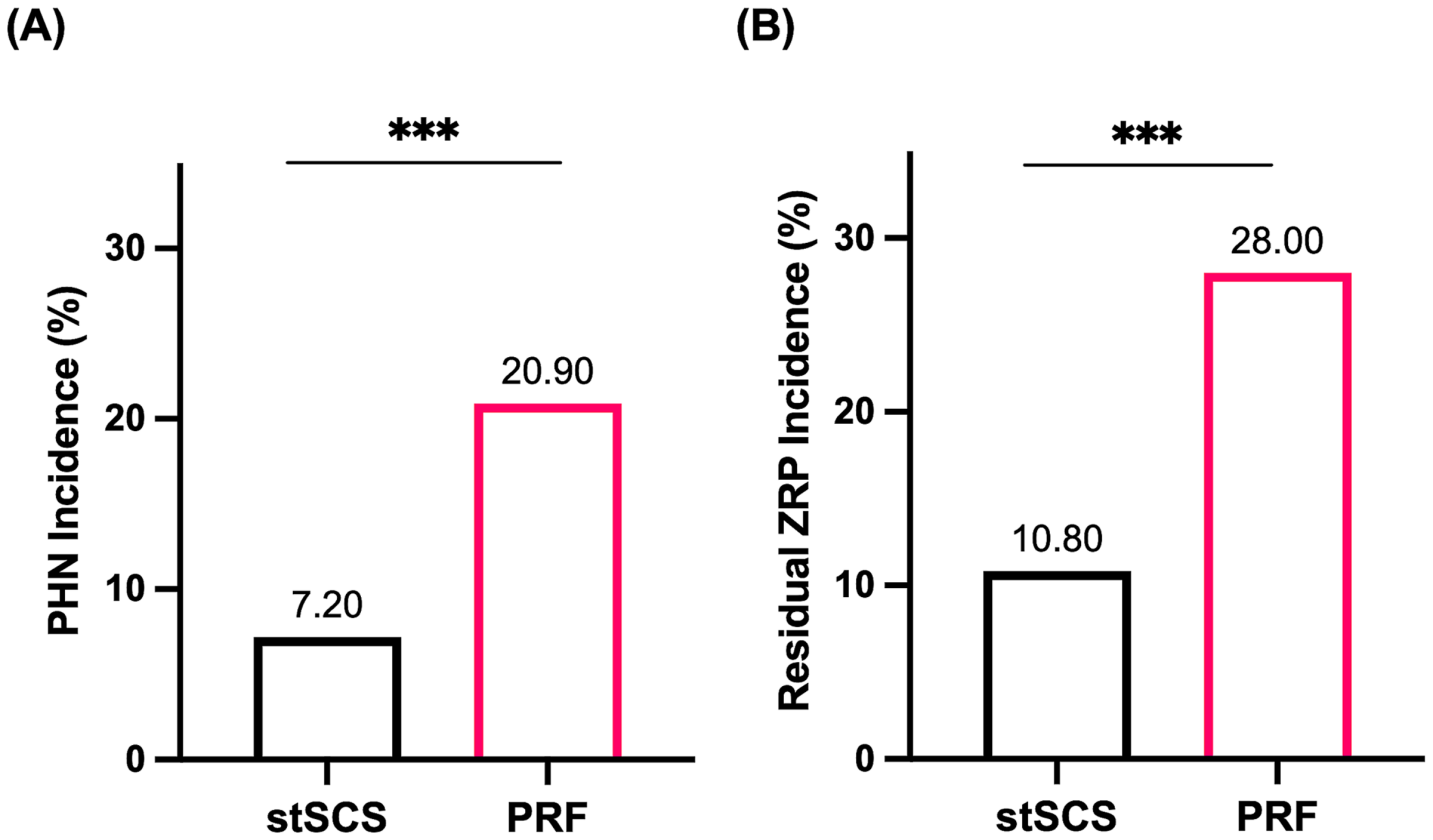
Incidence of postherpetic neuralgia (PHN) and residual zoster-related pain (ZRP) in the stSCS and PRF groups. **(A)** PHN was defined as persistent pain with a NRS score ≥ 3 beyond 90 days after herpes zoster onset. The incidence of PHN was 7.2% (12/167) in the stSCS group and 20.9% (53/254) in the PRF group. **(B)** Residual ZRP was defined as persistent pain with an NRS score ≥ 1 beyond 90 days after herpes zoster onset. The incidence of residual ZRP was 10.8% (18/167) in the stSCS group and 28.0% (71/254) in the PRF group. ***p < 0.001 for the between-group comparison.

As shown in Table 3 and illustrated in Figure 4, the incidence of residual ZRP was significantly lower in the stSCS group than in the PRF group (10.8% vs. 28.0%, OR = 0.31, 95% CI, 0.18–0.55, *p* < 0.001). The incidence of PHN remained lower in the stSCS group than in the PRF group (7.2% vs. 20.9%, OR = 0.29, 95% CI, 0.15–0.57, *p* < 0.001). These findings suggest that early application of stSCS not only provides superior short- and mid-term pain relief but also was associated with a lower risk of progression to persistent neuropathic pain.

To account for the nonrandomized treatment allocation, multivariable logistic regression analyses were performed for persistent pain outcomes. As shown in Table 3, after adjustment for age, sex, BMI, disease duration, disease phase, baseline NRS score, affected dermatome, and major comorbidities, stSCS remained independently associated with a lower risk of residual ZRP compared with PRF (adjusted OR = 0.35, 95% CI: 0.18–0.70; *p* = 0.002). Similarly, the association between stSCS and lower clinically significant PHN incidence remained directionally consistent after adjustment (adjusted OR = 0.30, 95% CI: 0.14–0.66; *p* = 0.003). These adjusted results support the robustness of the primary findings, although causal inference remains limited by the retrospective observational design.

### 3.5 Quality of life

Both interventions were associated with significant improvements in sleep quality and functional status over time (Figures 3B, C and Table 4). As shown in Table 4, AIS scores, reflecting sleep disturbance severity, decreased markedly in both groups following treatment. Patients in the stSCS group demonstrated a more pronounced reduction in AIS scores, with median values decreasing from 12.0 (9.0, 15.0) at baseline to 7.0 (5.0, 9.0) at 2 weeks, 3.0 (2.0, 5.0) at 1 month, and 1.0 (1.0, 3.0) at 3 months. In comparison, the PRF group showed a more modest improvement, with AIS scores declining from 12.0 (8.0, 15.0) at baseline to 10.0 (6.0, 13.0), 6.0 (4.0, 8.0), and 4.0 (2.0, 6.0) at the corresponding time points. Between-group differences in AIS scores were statistically significant at all post-treatment time points (all *p* < 0.001).

**Table 4:** Changes in AIS and ADL scores before and after treatment.

Indicator	Time point	stSCS group	PRF group	*p*-value*
AIS score	Baseline	12.0 (9.0, 15.0)	12.0 (8.0, 15.0)	0.092
	2 weeks	7.0 (5.0, 9.0)	10.0 (6.0, 13.0)	<0.001
	1 month	3.0 (2.0, 5.0)	6.0 (4.0, 8.0)	<0.001
	3 months	1.0 (1.0, 3.0)	4.0 (2.0, 6.0)	<0.001
ADL score	Baseline	65.0 (60.0, 70.0)	65.0 (60.0, 70.0)	0.451
	2 weeks	80.0 (70.0, 85.0)	80.0 (75.0, 85.0)	0.506
	1 month	85.0 (80.0, 90.0)	85.0 (80.0, 90.0)	0.830
	3 months	90.0 (85.0, 100.0)	90.0 (85.0, 100.0)	0.639

Data are presented as median (interquartile range). For post-treatment between-group comparisons of AIS and ADL scores, Bonferroni correction was applied across three follow-up time points for each outcome, with an adjusted significance threshold of 0.0167. *P-values indicate between-group comparisons at each time point. Within-group significance over time is illustrated in Figures 3B,C; AIS scores decreased significantly and ADL scores increased significantly from baseline at all post-treatment time points in both groups (all *P* < 0.001).

AIS, Athens Insomnia Scale; ADL, Activities of Daily Living.

In contrast, ADL scores improved significantly over time in both groups, indicating enhanced functional independence. Median ADL scores increased from 65.0 (60.0, 70.0) at baseline to 80.0 (70.0, 85.0) at 2 weeks, 85.0 (80.0, 90.0) at 1 month, and 90.0 (85.0, 100.0) at 3 months in the stSCS group. Similar improvements were observed in the PRF group, with no statistically significant differences between groups at any follow-up time point (all *p* > 0.05).

### 3.6 Subgroup analyses

**Figure 5:**
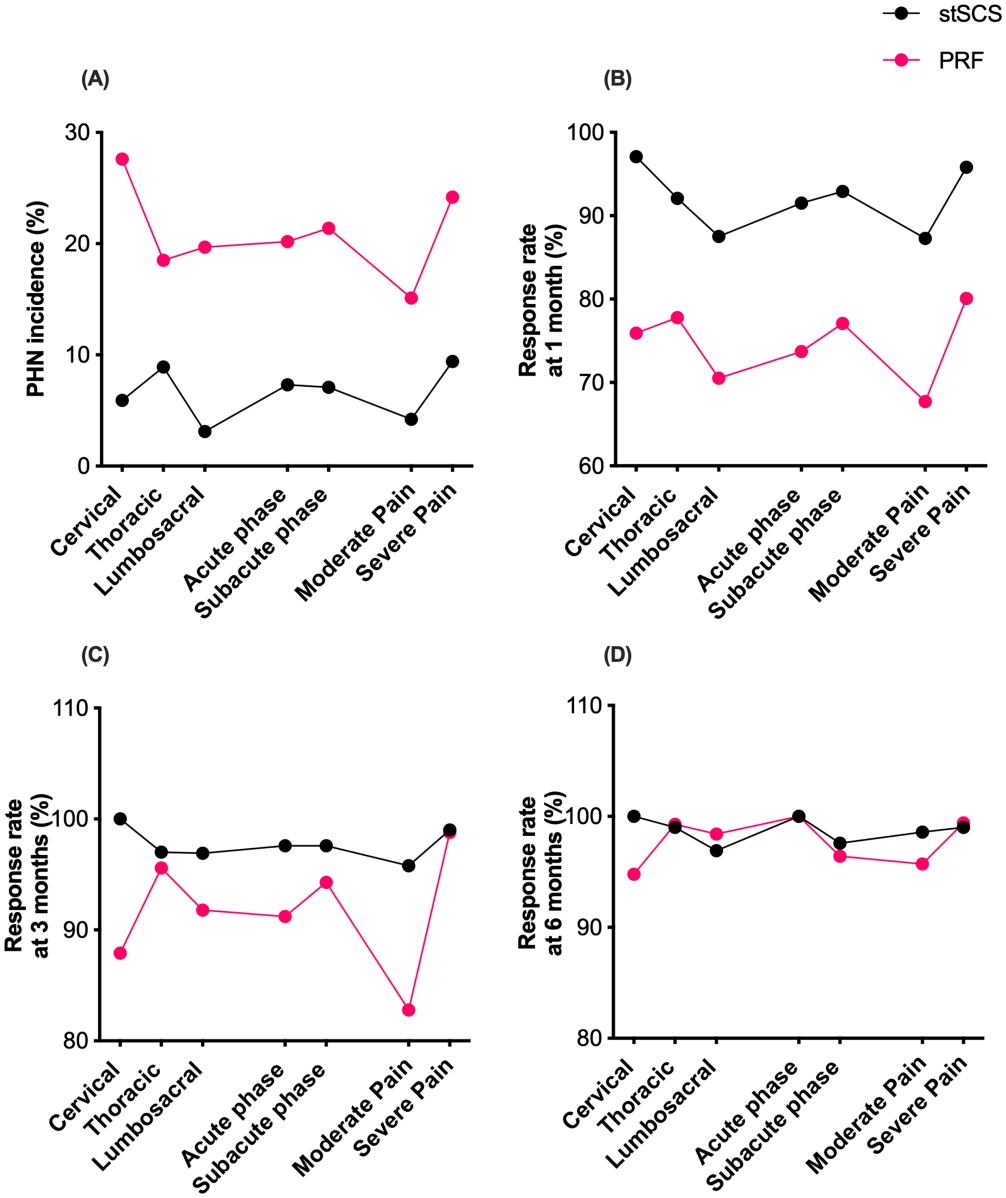
Subgroup interaction plots of treatment outcomes in the stSCS and PRF groups. **(A)** PHN incidence according to dermatome, disease phase, and baseline pain severity. **(B)** Responder rate at 1 month according to dermatome, disease phase, and baseline pain severity. **(C)** Responder rate at 3 months according to dermatome, disease phase, and baseline pain severity. **(D)** Responder rate at 6 months according to dermatome, disease phase, and baseline pain severity. Subgroups were defined by dermatome distribution (cervical, thoracic, and lumbosacral), disease phase (acute and subacute), and baseline pain severity (moderate and severe).

Subgroup analyses were performed according to dermatome level, disease phase, and baseline pain severity to evaluate the consistency of treatment effects between stSCS and PRF. Treatment effects were visualized using interaction plots (Figures 5A–D), and statistical heterogeneity was assessed using the Breslow–Day test.

As shown in Figures 5A–D and summarized in Tables 5–7, stSCS showed a generally favorable direction of effect, with lower PHN incidence and higher responder rates than PRF across the examined subgroups. Formal interaction testing revealed no significant heterogeneity of treatment effects by dermatome, disease phase, or baseline pain severity for all evaluable comparisons (all interaction p > 0.05). For the 6-month response rate by disease phase, interaction testing was not applicable because responder rates approached ceiling levels in both groups.

**Table 5:** Treatment effects by dermatome.

Indicator	Group	PHN incidence	Response rate (1 month)	Response rate (3 months)	Response rate (6 months)	
Cervical	stSCS	2/34 (5.9%)	33/34 (97.1%)	34/34 (100%)	34/34 (100%)	
	PRF	16/58 (27.6%)	44/58 (75.9%)	51/58 (87.9%)	55/58 (94.8%)	
Thoracic	stSCS	9/101 (8.9%)	93/101 (92.1%)	98/101 (97.0%)	100/101 (99.0%)	
	PRF	25/135 (18.5%)	105/135 (77.8%)	129/135 (95.6%)	134/135 (99.3%)	
Lumbosacral	stSCS	1/32 (3.1%)	28/32 (87.5%)	31/32 (96.9%)	31/32 (96.9%)	
	PRF	12/61 (19.7%)	43/61 (70.5%)	56/61 (91.8%)	60/61 (98.4%)	
Interaction p^*^		0.363	0.528	0.290	0.350	

Data are presented as number/total number (%). Interaction *p*-values were calculated using the Breslow–Day test to assess heterogeneity of treatment effects between the stSCS and PRF groups across dermatome subgroups (cervical, thoracic, and lumbosacral).

PHN, postherpetic neuralgia.

**Table 6:** Treatment effects by disease phase.

Indicator	Group	PHN incidence	Response rate (1 month)	Response rate (3 months)	Response rate (6 months)
Acute	stSCS	6/82 (7.3%)	75/82 (91.5%)	80/82 (97.6%)	82/82 (100%)
	PRF	23/114 (20.2%)	84/114 (73.7%)	104/114 (91.2%)	114/114 (100%)
Subacute	stSCS	6/85 (7.1%)	79/85 (92.9%)	83/85 (97.6%)	83/85 (97.6%)
	PRF	30/140 (21.4%)	108/140 (77.1%)	132/140 (94.3%)	135/140 (96.4%)
Interaction p^*^		0.646	0.768	0.870	/ ^#^

Data are presented as number/total number (%). Interaction *P*-values were calculated using the Breslow–Day test to assess heterogeneity of treatment effects between the stSCS and PRF groups across disease phases (acute vs. subacute). At the 6-month follow-up, interaction testing for responder rates was not applicable because responder rates approached ceiling levels in both treatment groups, resulting in insufficient variability for reliable estimation of interaction effects.

PHN, postherpetic neuralgia.

**Table 7:** Treatment effects by baseline pain severity.

Indicator	Group	PHN incidence	Response rate (1 month)	Response rate (3 months)	Response rate (6 months)
Moderate (NRS 3–6)	stSCS	3/71 (4.2%)	62/71 (87.3%)	68/71 (95.8%)	70/71 (98.6%)
	PRF	14/93 (15.1%)	63/93 (67.7%)	77/93 (82.8%)	89/93 (95.7%)
Severe (NRS 7–10)	stSCS	9/96 (9.4%)	92/96 (95.8%)	95/96 (99.0%)	95/96 (99.0%)
	PRF	39/161 (24.2%)	129/161 (80.1%)	159/161 (98.8%)	160/161 (99.4%)
Interaction p^*^		0.347	0.779	0.303	0.337

Data are presented as number/total number (%). Interaction *P*-values were calculated using the Breslow–Day test to assess heterogeneity of treatment effects between the stSCS and PRF groups according to baseline pain severity (moderate vs. severe).

NRS, Numeric Rating Scale; PHN, postherpetic neuralgia.

The observed association between stSCS and lower PHN incidence was generally consistent across cervical, thoracic, and lumbosacral dermatomes, as well as in both acute and subacute disease phases. Similarly, higher short-term responder rates with stSCS were consistently observed across all subgroup strata. By 6 months, responder rates approached ceiling levels in both groups, with minimal between-group differences, suggesting convergence of long-term outcomes.

Overall, these analyses support the robustness and generalizability of the clinical benefits of stSCS over PRF, without evidence of clinically meaningful effect modification by anatomical distribution, disease stage, or baseline pain intensity.

### 3.7 Complications

No severe complications were observed. In the stSCS group, two patients experienced transient lead migration and one patient reported mild incision site discomfort, these events resolved with conservative management, lead repositioning, or local wound care as appropriate. In the PRF group, three patients developed pneumothorax after thoracic DRG procedures. All pneumothorax cases were mild and clinically stable, resolved with conservative treatment and observation, and did not require chest tube drainage or surgical intervention. The overall incidence of documented complications was 1.8% (3/167) in the stSCS group and 1.2% (3/254) in the PRF group (*p* = 0.603).

## 4 Discussion

In this study, we compared the clinical efficacy of stSCS and PRF in patients with acute or subacute zoster-related pain. Both interventions resulted in significant improvements in pain intensity, sleep quality, and ADL. However, stSCS demonstrated clear advantages in early analgesia, higher short- and mid-term responder rates, and a significantly reduced incidence of PHN/residual ZRP, supporting its potential role as an early neuromodulatory strategy.

### 4.1 Early analgesic superiority of stSCS

A key finding of this study is the more rapid and pronounced pain reduction achieved with stSCS, particularly within the first month following treatment. This early benefit is clinically meaningful, as the acute and subacute phases represent a critical window during which persistent nociceptive input may drive central sensitization. The possible analgesic mechanisms of stSCS include activation of large-diameter dorsal column fibers consistent with segmental gate-control principles, suppression of dorsal horn hyperexcitability and wide-dynamic-range neuronal firing, enhancement of inhibitory neurotransmission and descending inhibitory pathways, and attenuation of microglial/neuroinflammatory amplification (10–12). In contrast, PRF is thought to exert its effects primarily through reversible modulation of injured afferent pathways and DRG excitability rather than continuous dorsal column neuromodulation, which may partly explain its comparatively weaker early analgesic impact (17, 18).

### 4.2 Association between early stSCS and lower residual ZRP/PHN incidence

Beyond pain relief, the lower incidence of PHN/residual ZRP observed in the stSCS group represents one of the most clinically meaningful findings of this study. Persistent zoster-related pain and PHN are driven by complex and multifactorial pathophysiological mechanisms, including neuroinflammation, microglial and astrocytic activation, loss of inhibitory interneurons, and maladaptive synaptic plasticity within spinal and supraspinal pain networks (19, 20). Early and effective suppression of nociceptive signaling may interrupt these processes, thereby reducing the likelihood of transition to chronic neuropathic pain.

Previous studies have consistently demonstrated the efficacy of stSCS in patients with established PHN, with improvements in pain control and quality of life (4). The present findings extend this body of evidence by suggesting that stSCS, when applied during the acute or subacute phase, may confer benefits that go beyond symptomatic relief and potentially reduce the risk of subsequent PHN. This temporal shift—from treatment of established chronic pain to earlier intervention—represents an important conceptual advance in the neuromodulation-based management of zoster-related pain.

Previous epidemiological studies have reported that approximately 5–30% of patients with HZ develop PHN overall, with higher rates in older adults and in patients with severe acute pain (2–4). In this context, the PRF-group incidence of 20.9% is compatible with a high-risk clinical cohort, whereas the stSCS-group incidence of 7.2% is lower than expected. The additional multivariable analyses further supported the robustness of the observed association between stSCS and lower risk of persistent pain outcomes. After adjustment for clinically relevant baseline variables, stSCS remained associated with a reduced risk of residual ZRP, and the association with clinically significant PHN was directionally consistent. However, because treatment allocation was not randomized, these findings should be interpreted as associations rather than definitive evidence of causality.

The apparent discrepancy between the 3-month PHN incidence and the high 6-month responder rate should be interpreted in light of the different definitions and time frames of these outcomes. Clinically significant PHN was defined as persistent pain with an NRS score ≥3 at ≥90 days after rash onset, whereas responder status was defined as a ≥ 50% reduction in NRS score from baseline. Therefore, a patient with substantial pain reduction from a high baseline score could still meet the responder criterion even if residual clinically meaningful pain persisted. In addition, some patients with pain at 3 months may have improved further by 6 months due to natural recovery, medication adjustment, or additional non-neuromodulatory treatments. Because records of crossover or rescue interventions between 3 and 6 months were incomplete, the 6-month responder-rate convergence should be regarded as descriptive rather than definitive evidence of equivalent long-term efficacy.

### 4.3 Insights from subgroup and interaction analyses

Subgroup analyses demonstrated that the beneficial effects of stSCS on responder rates and PHN incidence were generally consistent across dermatomal distributions, disease phases, and baseline pain severity, with no statistically significant interactions detected. These findings support the robustness and generalizability of stSCS efficacy within the acute–subacute disease spectrum and across different anatomical and clinical presentations.

When stratified by baseline pain severity, stSCS was associated with numerically lower PHN incidence and higher responder rates than PRF in both moderate and severe pain subgroups. However, formal interaction testing did not reveal significant effect modification by baseline pain intensity. This suggests that, within the present cohort, the relative advantage of stSCS over PRF was broadly comparable across different levels of initial pain severity rather than being confined to patients with severe baseline pain. This observation differs from some prior reports proposing a preferential benefit of stSCS in patients with more severe pain (21). Potential explanations include differences in study design, patient populations, definitions of pain severity, and, importantly, the timing of intervention. The early application of neuromodulation in the present study may have mitigated pain severity–dependent differences by intervening before central sensitization became firmly established. In addition, relatively high responder rates at later follow-up time points may have limited the ability to detect interaction effects due to ceiling phenomena.

Overall, these findings suggest that early stSCS provides consistent benefits over PRF across clinically relevant subgroups, supporting its use as a broadly applicable neuromodulatory option rather than one restricted to patients with the most severe initial pain.

### 4.4 Quality-of-life outcomes and safety

Quality-of-life outcomes revealed a distinct advantage of stSCS in sleep improvement, whereas functional recovery (ADL) was similar between groups. Given the strong bidirectional relationship between sleep disturbance and chronic pain, the superior impact of stSCS on sleep may further contribute to its role in PHN prevention (22). Our observation that stSCS leads to greater improvements in AIS mirrors findings from the meta-analysis showing improved sleep quality (15). Such multidimensional benefits underscore the neuromodulatory advantages of stSCS beyond mere analgesia.

Both interventions showed favorable safety profiles, with only minor, transient adverse events reported. This is consistent with prior studies in elder and PHN cohorts demonstrating low rates of serious complications for both PRF and stSCS (23). Transient lead migration and mild incision discomfort after stSCS were minor and device-management related, whereas pneumothorax after thoracic PRF is a potentially serious procedure-specific complication that requires prompt recognition and monitoring. Therefore, clinical decision-making should weigh not only the overall complication rate but also the severity and manageability of specific adverse events, anatomical targets, operator experience, and patient comorbidity.

### 4.5 Clinical implications

Although both stSCS and PRF have been used in the management of zoster-related pain, the present study provides several clinically relevant insights. Most importantly, our findings support consideration of earlier neuromodulation, particularly stSCS, in patients with acute or subacute zoster-related pain who are at increased risk of persistent pain or PHN. Rather than reserving neuromodulation for refractory chronic cases, early stSCS may offer advantages in terms of faster pain relief and a lower likelihood of progression to chronic neuropathic pain.

Our results are consistent with a prospective randomized trial demonstrating lower NRS scores and reduced analgesic consumption with stSCS compared to PRF at short- and mid-term follow-up (14), as well as with a meta-analysis reporting superior pain relief, sleep improvement, and fewer adverse events with stSCS (15). Taken together, these findings support a mechanism-based treatment algorithm in which stSCS may be considered early for patients with severe acute/subacute ZRP or elevated PHN risk, while PRF remains a reasonable alternative when patients prefer a shorter and less invasive procedure, when implantation resources are unavailable, or when the target DRG can be treated safely. Recent evidence has also suggested that outcomes after radiofrequency-based interventions for herpes zoster-related pain may depend on the specific technique used, with recurrence risk differing across procedures, which further highlights the importance of selecting an intervention not only on the basis of short-term pain control but also on expected durability of benefit (24, 25).

### 4.6 Limitations

This study has several limitations. First, its retrospective single-center design may introduce selection bias and residual confounding, and the nonrandomized treatment allocation precludes causal inference. Second, although multivariable logistic regression was performed to adjust for measured baseline covariates, residual confounding from unmeasured factors remains possible. Third, 6-month outcomes may have been influenced by unrecorded concomitant treatments during follow-up. Finally, cost-effectiveness was not assessed. Prospective multicenter randomized studies with standardized follow-up and adjusted analyses are needed to confirm these findings.

## 5 Conclusion

Our findings corroborate existing evidence that stSCS offers superior analgesia, improved sleep and function, and reduced PHN/residual ZRP risk compared to PRF in the management of acute/subacute zoster-related pain. These outcomes support incorporating stSCS earlier in neuropathic pain management algorithms. However, prospective controlled trials and economic analyses are needed to further validate its role in clinical practice. These findings echo and extend insights from randomized controlled trials and meta-analyses on the topic.

## Data Availability

The raw data supporting the conclusions of this article will be made available by the authors, without undue reservation.
